# Female doping: observations from a data lake study in the Hospital District of Helsinki and Uusimaa, Finland

**DOI:** 10.1186/s12905-023-02399-9

**Published:** 2023-05-09

**Authors:** Paula Katriina Vauhkonen, Teemu Daniel Laajala, Katarina Mercedes Lindroos, Mikko Ilari Mäyränpää

**Affiliations:** 1 Forensic Medicine Unit, Finnish Institute for Health and Welfare, P.O. Box 30 (Mannerheimintie 166), 00271 Helsinki, Finland; 2 Faculty of Medicine, University of Helsinki, P.O. Box 63 (Haartmaninkatu 3), 00014 Helsinki, Finland; 3 Department of Mathematics and Statistics, University of Turku, Yliopistonmäki (Vesilinnantie 5), 20014 Turku, Finland; 4 Department of Pathology, University of Helsinki, P.O. Box 21 (Haartmaninkatu 3), 00014 Helsinki, Finland; 5 Helsinki University Hospital, P.O. Box 340, FI-00029 Helsinki, Finland

**Keywords:** Doping, Anabolic androgenic steroids, Substance abuse, Morbidity, Specialized health care, Women

## Abstract

**Background:**

Doping is a well-recognized risk factor for several potentially severe health effects. Scientific literature concerning the need for medical treatment for such adversities is still sparse. This is especially true for women, due to lower doping use prevalence compared to men. Our study explored the nature of medical contacts and deviance in red blood cell parameters of female patients with doping use in Finnish specialized health care.

**Methods:**

This was a retrospective register study. The study sample was gathered from the Hospital District of Helsinki and Uusimaa, Finland (HUS) Datalake. An exhaustive search for doping related terms was performed to find patients with doping use documentation within free-text patient records. Medical record data was supplemented with laboratory data and medical diagnoses covering a total observation time of two decades. Statistical analysis included Fisher's Exact Test and one-way ANOVA.

**Results:**

We found 39 female patients with history of doping use and specialized health care contacts in the HUS-area between 2002–2020. At initial contact (i.e., the first documentation of doping use), the mean age of these patients was 33.6 years (min 18.1, max 63.5, SD 10.6). The most frequently used doping agents were anabolic androgenic steroids (AAS). The initial contacts were significantly more often acute in nature among patients with active doping use than among patients with only previous use (no use within one year; *p* = 0.002). Psychiatric and substance use disorder (SUD) morbidity was high (46.2% and 30.8%, respectively). Eight patients (20.5%) had received specialized health care for acute poisoning with alcohol or drugs, and nine (23.1%) for bacterial skin infections. Less than 45% of patients with active AAS use presented with off-range red blood cell parameters.

**Conclusions:**

Our findings suggest that female patients with a history of doping use encountered in specialized health care may exhibit high psychiatric and SUD related morbidity. Also, majority of patients with AAS use had red blood cell parameters within-range. Further studies are required to assess the generalizability of these findings to patients within primary health care services, and to determine the usefulness of hematological parameters as indicators of AAS use in female patients.

**Supplementary Information:**

The online version contains supplementary material available at 10.1186/s12905-023-02399-9.

## Background

Modern doping and the wide-spread use of anabolic androgenic steroids (AAS) among elite athletes began in the 1950’s. The blossoming of gym culture in the 1970’s paved the way for gradual spread of doping substances outside competitive sports [1, 2]. At present, doping can be defined as an act that aims to enhance not only physical performance but also intellectual capability or appearance, by using any variation of substances classified as APEDs (appearance- and performance-enhancing drugs) or PIEDs (performance and image enhancing drugs). AAS are still the most commonly used PIEDs, but other important drugs in this category include (but are not limited to) non-steroidal anabolics (insulin, insulin-like growth hormone (IGF), human growth hormone (HGH) and beta-2 adrenergic drugs); diuretics; ergo/thermogenics (e.g., ephedrine, levothyroxine (T4), triiodothyronine (T3)); nootropics or “cognition enhancers” for brain doping (e.g., modafinil, dexamphetamine); and skin tanners (e.g., afamelanotide and other synthetic forms of melanocyte-stimulating hormone (MSH)). Also, common drugs of abuse (stimulants, depressants, opioids and cannabinoids) may be used for doping purposes [2, 3]. From a public health point of view, it is important to understand that doping usage trends reflect the current body ideal in society [2]. Following the “fitness revolution” in the 90’s, the ideal female figure has changed from thin to athletic, and a toned, muscular body is now preferred more than before [2, 4]. In this pursuit for perfection, females may also resort to AAS and other PIEDs [5, 6].

So far, epidemiological estimates indicate low prevalence of doping use among women in the general population: In a comprehensive meta-analysis published in 2014, the global lifetime prevalence of AAS use was estimated at 3.3%; 6.4% for males and 1.6% for females [7]. Recent national drug surveys indicate that 0.3 – 0.4% of Finnish women have used doping substances [8–10] *and personal communication with K. Karjalainen, Senior Researcher at Finnish Institute for Health and Welfare (THL), 7 Feb, 2022*. At the same time, the use of doping substances is markedly prevalent among certain populational subgroups. Studies of female bodybuilders have yielded 9.1% – 17.8% lifetime prevalence of AAS use globally, and the risk is also heightened among sexual and gender minorities and people with SUDs [11–14]. These findings may reflect polypharmacy practiced in connection with AAS use [6, 15–18]. Some people with doping use also develop a SUD regarding AAS or other PIEDs [16, 19].

The relationship between PIEDs use and psychopathology in females is not well-established. A wide range of psychological symptoms (including mood change, anxiety and insomnia) are frequently reported in connection with AAS use [17, 20, 21]. Hypomanic symptoms – often perceived as a positive side effect – may emerge during use, while depression is reported after discontinuation of use [15, 22]. Moreover, a recent study of female weightlifters by Scarth et al. found that those with current or previous AAS use demonstrated significantly greater psychopathology than those without AAS use [23]. On the other hand, females may be less prone to AAS use related aggression and psychological distress than males [24]. In some instances, PIEDs use merely reflects disordered weight management behavior, such as anorexia or muscle dysmorphia [25].

Regarding somatic morbidity, cardiovascular health has been of special concern. The use of AAS promotes an overall prothrombotic state by stimulating erythropoiesis, thrombocyte aggregation and blood coagulation factors in a dose-dependent manner [26]. AAS use also causes proatherogenic changes in the serum lipid profile [27] and blood pressure [28]. Long-term AAS exposure is associated with left ventricular dysfunction and acceleration of coronary atherosclerosis [29]. Other well-known severe adversities relate to liver toxicity, especially concerning orally administrated 17-α alkylated anabolic steroids. Cases of renal injury have been reported as well [27]. In addition, injecting AAS and other PIEDs may cause cutaneous bacterial infections and predispose to transmission of blood-borne pathogens (e.g., hepatitis B, C and HIV), if needles or syringes are shared [30].

Finally, AAS use may have profound effects on reproductive organs. In females, the androgenic properties of these agents cause virilization: irregularities in menstrual cycle, hirsutism, androgenic alopecia, enlargement of clitoris (clitoromegaly), reduced breast volume and deepening of voice [20, 31]. Dysphonia and clitoromegaly often persist after cessation of use [31, 32], and may require surgical treatment.

Current scientific literature concerning long-term morbidity relating to doping use is highly concentrated on males. These studies have found increased cardiovascular and psychiatric morbidity, increased annual number of hospital contacts and premature death among AAS using males, compared to AAS-negative peers or populational controls [22, 33, 34]. In a 30-years follow-up study of male former power sports athletes, those with prior AAS use self-reported higher lifetime prevalence of seeking professional expertise for depression, anxiety and tendon ruptures than those with no prior AAS use, while the lifetime prevalence of several other disease categories (including cardiovascular disease) did not differ between the groups [35]. In a study by Gruber & Pope [15], both AAS-using (*n* = 25) and non-using (*n* = 50) women athletes exhibited similar lifetime rates of psychiatric Axis I DSM-IV disorders. None of those with AAS use (aged 31.0 ± 5.9 years) in this study reported medical history of cardiovascular disease. In another study by Ip et al. [16], a web-based survey managed to reach 242 women of whom 12 had history of AAS use. These women reported significantly higher frequencies of prior psychiatric diagnoses than men with AAS use or women with no AAS use (50.0% vs. 17.4% and 22.2%, respectively), and exhibited higher rates of substance dependence symptoms regarding PIEDs use.

The real-world morbidity of females with doping use requires further investigation. Current knowledge on the health issues is scarce, and largely based on self-reporting. Furthermore, it is unclear, which of these doping-associated medical conditions or diseases require treatment in public health care. The primary objective of this study was to explore the medical diagnoses of doping using female patients in public specialized health care, and the timely relationship between these diagnoses and active doping use. Since AAS use may deviate hematological parameters, our second objective was to determine, whether variation in these parameters would correlate with the patients’ doping use status and thus be considered as an indicator of active use and consequently, increased thromboembolic complication risk. Our fundamental aim was to gather information that would help clinicians recognize female doping use better and raise awareness of the urgent and non-urgent conditions these patients may have. To our knowledge, this is the first study utilizing health care registers for exploring morbidity of females with doping use.

## Methods

### Data acquirement

**Fig. 1:**
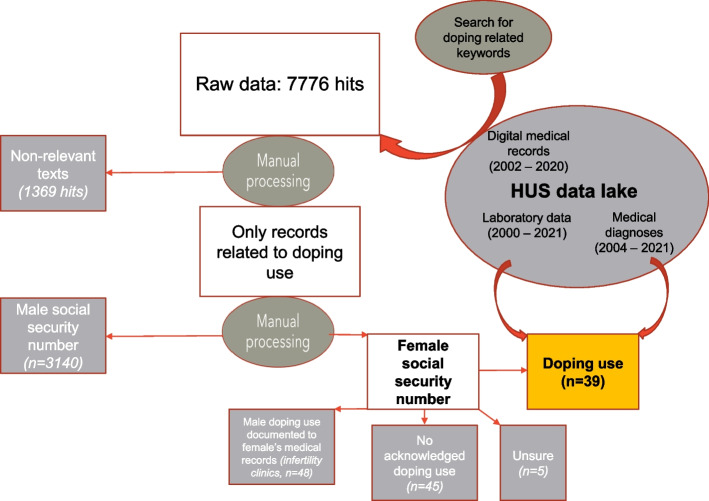
Description of the data acquirement process

The data acquirement process is visualized in Fig. 1.

The study sample was gathered from the Hospital District of Helsinki and Uusimaa, Finland (HUS) datalake, in collaboration with HUS Data Service. HUS datalake is a CE certified big data depository that receives data from over 100 different databases and covers 2–6 million electronic medical records per year from public health care hospitals and outpatient clinics in the HUS area from the year 2002 onwards [36]. HUS is the largest hospital district in Finland, the population in the special catchment area (2.2 million) covering nearly 40% of the Finnish population. Data collection was executed by searching for doping related keywords in the datalake’s digital medical records. These included expressions commonly used in the Finnish language for doping (including recreational and brain doping), as well as doping related laboratory tests. More detailed description of the used keywords is provided in an additional file (see Additional file 1).

The search produced originally 7776 patient records with one or more doping related keywords. The data was delivered as a.csv file containing the exact sentence in which the doping related term existed, and the sentences around it (one before and one after). The date, time and outpatient clinic or ward in question were provided alongside the free text. Non-relevant texts were screened out manually. Most of these concerned medicinal use of anabolic steroids, principally cachexia related to chronic disease. For cases that could not be reliably classified based on the available text (due to typographical errors or inexact documentation), the entire free text including the keyword was acquired (*n* = 291). After excluding all non-relevant texts, the cases were divided into males and females based on social security number, which in Finland includes the information about the patient’s birth date and sex. Some texts from infertility clinic were documented for the male and some for the female of the couple in question, and only in one case the text referred to female doping use. The rest of the cases were excluded from this study as they referred to male doping use.

### Processing of medical record data

Medical record data was manually reviewed by three researchers in order to reach a consensus on the correct doping status classification. Only patients with ascertained doping use were included in the study sample. Cases where the patient herself had not acknowledged doping use were left out of further analysis (*n* = 45), because none of the females had supporting tests (such as urinary AAS assay) performed. Five cases were excluded because their doctor had only speculated about possible doping use, but the patient herself was evidently never confronted with this question.

The first time that doping use was documented in the patient records was considered as “initial contact”. Doping use status of initial contact was further classified as ‘active’ (current use or use within one year) or ‘former’ (no use within one year). In case of inconsistent doping use history documentation, doping use status was declared ‘unsure’.

### Processing of medical diagnoses

All the medical diagnoses in the study are based on the 10th revision of the International Statistical Classification of Diseases and Related Health Problems (ICD-10), which is the revision used in Finland since 1996. Medical diagnoses within the datalake (available from the year 2004 onwards) were combined with medical record data, including initial contact diagnoses and all available diagnoses prior to and after the initial contact. The medical conditions of initial contact were further categorized as acute or non-acute, based on the main ICD-10 diagnosis and the contact type (emergency ward or in-patient care indicating acute conditions and outpatient care non-acute conditions).

### Processing of laboratory data

Laboratory test results (available from the year 2000 onwards) were categorized into 5 phases, based on the anamnestic timeline of doping use. Phase 0 represent laboratory tests taken before reported doping initiation; phase 1 during active doping use; phase 2 within three months of doping use cessation; phase 3 three to six months after of doping use cessation; and phases 4 and 5 later laboratory tests taken from patients ≤ 40 years old (phase 4) or older (phase 5) at the time of the measurement. Since menarche and the onset of cyclic variation of sex hormone levels may cause alterations in the red blood cell parameters [37], an age limit of 15 years was set for phase 0 laboratory results and phases 4 and 5 were separated according to the earliest onset of normal menopausal transition [38]. Phase 2 and 3 laboratory results were included only for those patients who were documented as having stopped using, whereas phase 4 and 5 laboratory results were included despite the possible continuation of use, in order to estimate the number of patients that may have continued or resumed doping use later in life.

The hematological parameters assessed in detail were hemoglobin concentration (Hb), erythrocyte count (RBC), hematocrit (HCT), and red blood cell indices (mean corpuscular volume (MCV), mean corpuscular hemoglobin (MCH), mean corpuscular hemoglobin concentration (MCHC) and red cell distribution width (RDW)). The patients’ medical diagnoses were compared with the laboratory test dates to exclude injury or disease that could have affected the parameters drastically (such as severe bleeding, cancer, or HIV). Additionally, patients with the diagnosis of transsexualism were left out of laboratory analysis because of the possible initiation of hormone treatment affecting the results.

### Statistical analysis

All analyses were performed using R Statistical Software, version 4.1.1 [39]. Differences in frequencies of count data were assessed using Fisher's Exact Test (two-tailed). For continuous variables, one-way ANOVA was performed. The alpha level for statistical significance was set at 0.05.

## Results

### Features of the study sample

**Fig. 2:**
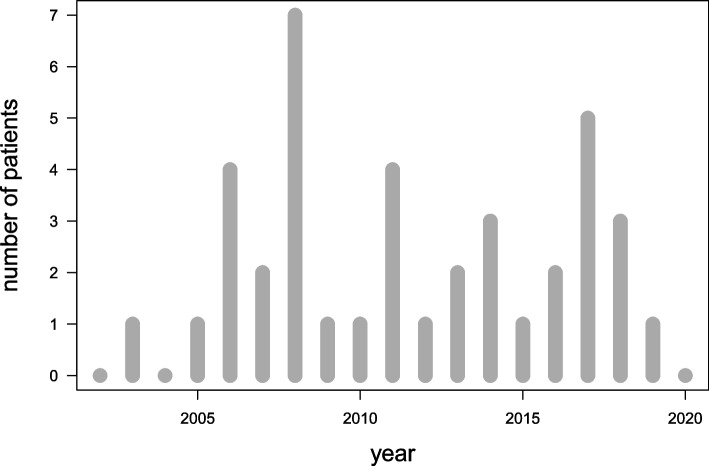
Annual frequencies of patients in HUS specialized health care (2002 – 2020)

The search yielded altogether 39 female patients with medical history of doping use, 0 – 7 new cases per year. There was a peak in 2008 (7 patients), whereas zero new patients were identified from 2002, 2004 or 2020 (Fig. 2).

Since doping use was often discussed with the patient on several occasions, there were on average three free text sections available per patient. The demographic and doping use characteristics of the study sample at initial contact are summarized in Table 1.

**Table 1:** Demographic and doping use characteristics of the study sample at initial contact

**Age (years), mean, min – max (SD)**	33.6, 18.1 – 63.5 (10.6)
**Age group (years)**, N (%)	
Under 20	2 (5.1)
20–24	8 (20.5)
25–29	7 (17.9)
30–34	6 (15.4)
35–39	5 (12.8)
40–44	5 (12.8)
45–49	4 (10.3)
Over 50	2 (5.1)
**Outpatient clinic/ward**, N (%)	
Internal medicine	11 (28.2)
Psychiatry	7 (17.9)
Gynecology	7 (17.9)
Surgery	5 (12.8)
Neurology	3 (7.7)
Pulmonology	2 (5.1)
Emergency	2 (5.1)
Phoniatrics	1 (2.6)
Ear, nose, and throat diseases	1 (2.6)
**Doping use status**^**a**^, N (%)	
Active	20 (51.3)
Former	12 (30.8)
Unsure	7 (17.9)
**Cumulative use (months)**^**b**^, median, min – max (IQR)	4.0, 1.0 – 132.0 (30.0)
**Documented reason for use**, N (%)	
Competitive sports	22 (56.4)
*Bodybuilding*	10 (25.6)
*Powerlifting*	5 (12.8)
*Fitness*	3 (7.7)
*Other sports (not specified)*	4 (10.3)
Other	17 (43.6)
*Gym*	6 (15.4)
*Weight loss*	2 (5.1)
*Gender incongruence*	2 (5.1)
*Not specified*	7 (17.9)
**Documented used substance**, N (%)	
AAS^c^	28 (71.8)
AAS + other substances	6 (15.4)
*stimulants*	2 (5.1)
*HGH*^d^	1 (2.6)
*diuretics*	1 (2.6)
*several other substances*	2 (5.1)
Only other than AAS	4 (10.3)
*stimulants*	2 (5.1)
*clenbuterol*	1 (2.6)
*meldonium*	1 (2.6)
Unknown (given by coach)	1 (2.6)

^a^Based on anamnestic information: active = current use or use within one year; former = no use within one year; unsure = inexact documentation or discrepancy with clinical findings

^b^Cumulative use was documented only for 13 patients (33.3%)

^c^Anabolic androgenic steroids

^d^Human growth hormone

At initial contact, 20 patients reported active doping use and 12 only former use. Seven patients’ doping use status was unsure. This was mainly due to inexact doping use history documentation; however, two patients acknowledged only former use, but their clinical findings repeatedly suggested they were still using. Patients with active doping use were slightly younger than those with only former use (mean age 32.3 years and 37.5 years, respectively), but there were no statistically significant differences in mean age between the three use status groups, including those with unsure use status (mean age 30.7 years) (*p* = 0.305, ANOVA).

Majority of the patients (56.4%) had used doping substances for competitive sports. In about one fourth of the cases (25.6%), the documented reason for use had no reference to competitive activity. These included working out at the gym, desire to lose weight, and gender incongruence (desire to “look like a boy” by two AAS using birth-assigned females).

The most frequently used doping agents were AAS, either alone (71.8%) or in combination with other substances (15.4%). Two patients had been taking several substances with AAS, including clenbuterol, HGH, insulin, T3 and T4. Only half of the patients with AAS use (17/34) had documentation of the exact substance within the patient records. The documented AAS were oxandrolone, nandrolone, methandrostenolone, methenolone enanthate, boldenone, mesterolone, stanozolol and testosterone. The exact dose was documented only for three patients: oxandrolone 10 mg per day; methandrostenolone 30 mg per day with weekly boosts of testosterone (250 mg) and boldenone (300 mg); and meldonium 500 mg per day. In addition, two patients reported taking “mild AAS, specifically oriented for females”; and one patient reported taking the same dose her boyfriend was taking.

Five patients (12.8%) had at some point described the following AAS related signs of virilization to their doctor: amenorrhea (*n* = 3), lowering of voice (*n* = 3), hirsutism (*n* = 1), hair loss (*n* = 1), widening of chin (*n* = 1), and clitoromegaly (*n* = 1). In four additional cases (10.3%), the doctor had documented objective marks of virilization in the patient records.

### Medical conditions of initial contact

The medical conditions of initial contact are presented in Table 2.

**Table 2:** Medical conditions of initial contact, presented as the main ICD-10 diagnoses

Specialty	Acute conditions^a^	Non-acute conditions^a^
**Internal medicine**	Mental and behavioral disorders due to use of alcohol, withdrawal state	HIV disease resulting in (persistent) generalized lymphadenopathy (*n* = 2)
	Hypoglycaemia, unspecified	Crohn disease
	Left ventricular failure, atrial fibrillation/flutter	Essential (primary) hypertension
	Toxic liver disease with acute hepatitis	
	Left sided colitis	
	Acute HIV infection syndrome	
	Oedema, unspecified	
**Psychiatry**	Non-organic psychotic disorder	Severe depressive episode without psychotic symptoms
		Depressive episode, unspecified; Emotionally unstable personality disorder
		Dissocial personality disorder; Emotionally unstable personality disorder
		Nonorganic sleep disorder, unspecified
		Transsexualism (*n* = 2)
**Gynecology**	Miscarriage (*n* = 2)	Female infertility, unspecified
	Tubulo-interstitial nephritis	Excessive and frequent menstruation with regular cycle
		Hirsutism
**Surgery**	Assault by sharp object, shock	Injury of muscle and tendon of hip (over three months earlier)
	Embolism and thrombosis of arteries of lower extremities	Sprain and strain involving anterior cruciate ligament of knee (over three months earlier)
	Hematemesis	
**Neurology**	Headache	Epilepsy, unspecified
	Tension-type headache	
**Emergency**	Poisoning by drugs	
	Anxiety disorder, unspecified; Panic disorder	
**Pulmonology**	Lipid pneumonia	Sleep apnoea
**Ear, nose, and throat diseases**		Dysphonia
**Phoniatrics**		Aphonia
**Total**^**b**^	**19**	**19**

^a^N is specified if the condition appeared in more than one patient

^b^ICD-10 diagnosis was not available for one patient

For one patient, ICD-10 diagnosis of initial contact was not available. Half of the initial contacts (19/38) were due to acute medical conditions. Acute medical conditions were significantly more common among patients with active doping use (*n* = 16/20, 80.0%) than among patients with only former use (*n* = 2/11, 18.2%) (*p* = 0.002, Fisher).

### Other medical conditions

Other medical conditions included all the main and secondary ICD-10 diagnoses set before, during, or after the patients’ initial contact. Because of the low number of patients, only those conditions equal to or greater than five patients/diagnosis group are presented.

Eighteen patients (46.2%) were diagnosed with at least one psychiatric diagnosis, other than a SUD, and 12 patients (30.8%) with a SUD (Table 3). SUD diagnoses were more common among patients that also had another psychiatric diagnosis (*n* = 9/18, 50.0%) than among patients that did not have another psychiatric diagnosis (*n* = 3/21, 14.3%) (*p* = 0.035, Fisher).

**Table 3:** Mental and behavioral disorder diagnoses (ICD10: F00 – F99) among the study sample

Psychiatric disorders	Number of patients	%
Schizophrenia, schizotypal and delusional disorders (F20 – F29)	5	12.8
Mood [affective] disorders (F30 – F39)	9	23.1
Neurotic, stress-related and somatoform disorders (F40 – F48)	12	30.8
Behavioral syndromes associated with physiological disturbances and physical factors (F50 – F59)		
*Eating disorders*	2	5.1
*Nonorganic sleep disorders*	2	5.1
Disorders of adult personality and behavior (F60 – F69)		
*Personality disorders*	6	15.4
*Gender identity disorders*	3	7.7
**Total**	**18**^**a**^	**46.2**^**b**^
**Mental and behavioral disorders due to psychoactive substance use**		
Alcohol (F10.1 – F10.9)	4	10.3
Opioids (F11.1 – F11.9)	1	2.6
Sedatives (F13.1 – F13.9)	1	2.6
Polysubstance (any combination of psychoactive substances or F19.1 – F19.9)	6	15.4
*Alcohol and one more substance*	2	5.1
*Alcohol and several other substances*	2	5.1
*Opioids and stimulants*	1	2.6
*Multiple drug use, not specified*	1	2.6
**Total**	**12**	**30.8**

^a^Total is less than the sum of the individual disorders because many of the patients were diagnosed with more than one disorder

^b^Total percentage of patients with any psychiatric disorder

Five patients had some psychiatric diagnosis set before doping initiation. Two patients received their first psychiatric diagnosis during active AAS and concurrent stimulant use (first episode psychosis and unspecified anxiety/panic disorder) and another two a few months after AAS use cessation (depression and mixed obsessional thoughts/acts and an adjustment disorder). Regarding the rest of the patients (9/18, 50.0%), all available psychiatric diagnoses dated several years after active use. Among these, three patients (7.7%) were diagnosed with transsexualism (classified in the ICD-11 as gender incongruence). Two of these patients were birth-assigned females but one was male, having gone through male-to-female surgical operation. Additionally, eight patients (20.5%) were at least once hospitalized for acute poisoning with alcohol or drugs; two of these patients did not have a concurrent SUD diagnosis.

Unusual number of cutaneous bacterial infections were also observed. Nine patients (23.1%) had received specialized health care at least once for an abscess (*n* = 6), bacterial cellulitis (*n* = 3) and/or erysipelas (*n* = 3). In majority of the patients (7/9, 77.8%), these infections occurred after the initial contact. The proportion of patients with these bacterial infections was significantly higher among those with a SUD diagnosis (*n* = 7/12, 58.3%) than among patients without a SUD diagnosis (*n* = 2/27) (*p* = 0.001, Fisher). Blood-borne viral infections were diagnosed in five patients (12.8%): hepatitis C (*n* = 2) and HIV (*n* = 3, one with concurrent hepatitis C).

Several patients were also diagnosed with at least one disease of the musculoskeletal system or connective tissue: seven (17.9%) with a soft tissue disorder (ICD-10: M70 – 79) and seven (17.9%) with a dorsopathy (ICD-10: M40 – M54). However, there was no clustering of specific conditions within these diagnosis groups. In addition, five patients (12.8%) were diagnosed with an injury of hip or thigh (ICD-10: S70 – 79).

Altogether seven patients (17.9%) were diagnosed with a cardiovascular disease (ICD-10: I00 – 99, excluding I33.0 and I60 – 69) during the observation time. Two AAS using patients (5.1%) were diagnosed with left ventricular hypertrophy and dilated cardiomyopathy at a young age (both 39 years). The other patient had also suffered multiple cerebral infarcts and presented with lower extremity arterial embolism at initial contact, the etiology of which was considered most likely to be AAS use. The cumulative AAS exposure was not documented for either of these patients, but the medical records suggested several years of use. Additionally, one non-AAS using patient presented initially with left ventricular failure and was diagnosed with an underlying coronary heart disease at the age of 61. Four more patients were later diagnosed with some cardiovascular disease: two with left ventricular and congestive heart failure (at the age of 53 and 48, respectively), the latter patient presenting with essential hypertension at initial contact; one with supraventricular tachycardia; and another with deep vein thrombosis, occurring several years after the initial contact. There were no medical records available regarding these later diagnoses, indicating that the possible continuation of doping use has not been addressed or documented in connection with these visits in specialized health care.

Regarding adversities related to virilization, only two patients presented with voice disturbance (aphonia and dysphonia) and one with hirsutism at initial contact. Despite the six more patients with documented marks of virilization, corresponding medical diagnoses were not observed within the study sample. One patient presented initially with unspecified infertility and another with excessive and frequent menstruation. However, diagnoses of irregular or absent menstruation were not observed. Also, in addition to the one patient with toxic liver disease and acute hepatitis at initial contact, no other hepatotoxic events or medical diagnoses indicating chronic kidney failure were detected.

### Laboratory parameters

**Fig. 3:**
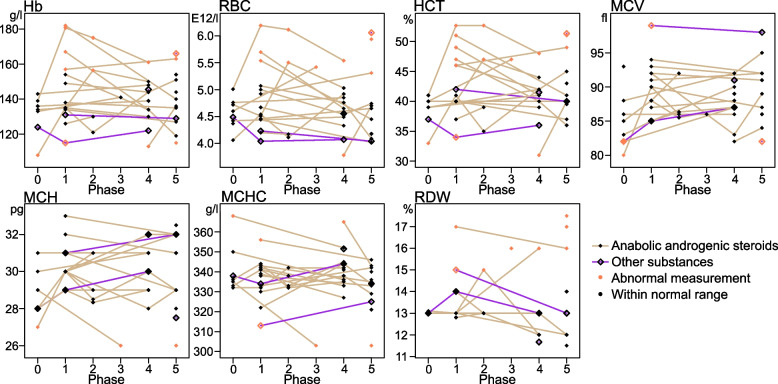
Trajectories of red blood cell parameters in different phases of doping use Phases represent the anamnestic timeline of doping use: 0 = before doping initiation; 1 = during active use; 2 = within three months of cessation; 3 = three to six months after cessation; 4 = later, despite possible continuation of use (patients aged ≤ 40 years); 5 = later, despite possible continuation of use (patients aged > 40 years). Individual patients had one laboratory measurement in each phase, except for phases 1 and 2 (two patients had two measurements) and phases 4 and 5 (one patient had two measurements). If more than one laboratory measurement was available for a phase, the symbol represents the mean value of these. The normal range for each measurement represents the reference range provided by the analyzing laboratory. Measurements of a single individual are connected with a solid line between phases, whenever possible. Patients with anabolic androgenic steroids use: n = 23 (solid diamond symbols/golden lines); Patients with use of only other substances: n = 4 (hollow diamond symbols/purple lines). Abbreviations: Hb = hemoglobin concentration, RBC = erythrocyte count, HCT = hematocrit, MCV = mean corpuscular volume, MCH = mean corpuscular hemoglobin, MCHC = mean corpuscular hemoglobin concentration, RDW = red cell distribution width

Red blood cell parameters were available and categorizable for 27 individual patients (Fig. 3).

During reported active doping use, HCT was above reference range in 41.7% (5/12), Hb in 33.3% (4/12), and RBC in 25.0% (3/12) of the patients with AAS use. Red blood cell indices were within limits, except for one patient with MCHC and another with RDW above range. Patients with no AAS use had Hb, RBC and HCT within or below the reference range during active use. Four patients with AAS use had laboratory measurements taken within three months of cessation. Three of these patients had normal range measurements during active use; two remained within limits after cessation, but one presented with deviant increase in Hb, RBC, HCT and RDW after discontinuation. The fourth patient remained clearly above Hb, RBC and HCT reference range. One patient with AAS use had laboratory measurements taken three to six months after reported cessation and presented with normal Hb but above range RBC and HCT, along with aberrant red blood cell indices. Phase 5 results indicate that at least three patients may have resumed AAS use later in life. Comparison of medical diagnoses revealed that only two AAS using patients had received the diagnosis of secondary polycythemia.

## Discussion

### Main findings of the study

This study was the first explorative study of female doping in the Finnish health care system. The data lake search yielded altogether 39 female patients with acknowledged doping use. The total number of discovered patients and observed annual frequencies are in line with the prevalence estimates of doping use among the Finnish population. Yet, the exact quantity of these patients in specialized health care services remains obscure. Previous studies indicate that the tendency to seek help from health care professionals is low among people who use AAS [40, 41]. The experienced side effects of AAS use may not be considered severe enough to require treatment, but these patients may also be reluctant to admit doping use when confronted by medical professionals, due to stigma or lack of trust in their doctor’s knowledge on the issue [21, 41]. Also, only partial truth may be served: in our study, two patients were willing to disclose only previous, not current use, despite conflicting clinical findings.

The initial documentation of doping use originated most often from the specialties of internal medicine, psychiatry, and gynecology. This may, in part, reflect the awareness of the problem in these special fields. Furthermore, although most of these patients were in their twenties or thirties, age was not a predictor of active doping use. The laboratory results also suggested that some of these patients may have continued or resumed AAS use later in life. Active doping use, in turn, was associated with the initial medical condition being acute and necessitating urgent evaluation. These findings highlight the importance of regarding doping use as one possible etiology for symptoms in patients of all ages.

While motivation for doping use was clearly linked to sports activities, three patients were diagnosed with gender incongruence and two birth-assigned females reported AAS use specifically for the desire to look more masculine. Indeed, previous studies indicate increased risk for nonprescription steroid use among transgender youth [42]. This behavior may reflect self-prescription of cross-sex hormones, more commonly encountered among birth-assigned males than females [43]. Our findings confirm that this behavior is not confined to trans women only.

Most importantly, we observed a high number of mental and behavioral disorders among the study sample. As demonstrated in epidemiological studies, there was significant comorbidity between SUDs and other psychiatric conditions also in our sample [44, 45]. In addition, we found an association between severe bacterial skin infections (including abscesses and cellulitis) and SUDs. This may indicate injection drug use and particularly the use of subcutaneous or intramuscular injections [46].

High rate of psychiatric illness has previously been reported in publications of both men and women with doping use [15, 16, 22, 23]. In our study, four patients had received their first psychiatric diagnosis during AAS use or within a few months of cessation. Otherwise, no temporal connection with reported doping use and psychiatric illness was observed. The percentage of patients with some psychiatric disorder was very similar to the study by Ip et al. [16], which also found these comorbidities to be more common among AAS using women than men. In the Swedish study of male former power sports athletes by Lindqvist Bagge et al. [35], only 18.2% of those with prior AAS use reported seeking professional expertise for psychiatric problems in their lifetime. Even though these studies are not straightforwardly comparable to ours, it does raise the question whether women with doping use suffer from even greater psychiatric morbidity than men. It is also possible that women seek professional help at a lower threshold than men [20, 21, 40].

Compared to mental and behavioral disorders, cardiovascular morbidity within the study sample was not as alarming. Two patients were diagnosed with left ventricular hypertrophy and dilated cardiomyopathy, the other with several hypercoagulable comorbidities, likely attributable to long term AAS use. Two patients were later diagnosed with heart failure and one with supraventricular tachycardia, but there was no documentation available regarding doping use continuation and other etiology cannot be excluded. Additionally, only one case of coronary heart disease and a single case of deep vein thrombosis was observed. Bearing in mind that our study sample represents specialized health care patients, these findings suggest that females with doping use may not be particularly susceptible to AAS related cardiovascular complications. Based on clinical studies, there is no increase in cardiovascular morbidity among females receiving low-dose testosterone therapy [47]. Thus, our findings may reflect lower doses, fewer substances, or shorter cumulative time of AAS use compared to males, consistently reported in several previous studies [16, 17, 20, 48]. The rationale behind this is perhaps sensitivity to androgenic adversities or fear of becoming too masculine, which is not considered ideal [16, 49].

As AAS induced increase in Hb and HCT may predispose to thromboembolic complications [26], we also investigated deviance in the red blood cell parameters of these patients in relation to reported doping use status. We found that less than 45% of patients with AAS use in our study presented with off-range red blood cell parameters during active use. Börjesson et al. [20, 48] have previously made similar observations in small samples of non-hospitalized AAS using females. These findings could explain the rarity of thromboembolic complications in our sample. Moreover, our results provide further support for the hypothesis that such parameters may not serve as a sensitive marker for AAS use in females.

We also observed several dorsopathies and soft tissue disorders among the study sample. This was expected, since people engaged with sports activities often suffer from musculo-skeletal strain. The high rate of hip and thigh injuries may, however, represent imbalance between muscle growth and tendon strength, as tendon ruptures have been associated with AAS use in some studies [26, 35].

Surprisingly, medical diagnoses relating to virilization were rare. This suggests that females with doping use are either not actively seeking help for these conditions, or these are only seldom recognized in specialized health care. It has also been previously noted that the person herself may not acknowledge AAS induced voice change, even when it is clearly observable by others [15]. Menstrual irregularities may, in turn, be viewed as a sign of effectivity of the used substances, rather than a feared side-effect [32].

One key observation was that lack of doping specific diagnoses in ICD-10 may render doping use invisible for other clinicians. Except for the two patients with secondary polycythemia (ICD-10: D75.1), there were no specific diagnoses that would have clearly indicated current or previous doping use. At the same time, verbal documentation is easily missed, especially with patients who use health care services regularly and accumulate heaps of medical records over the years.

Finally, data lake digital medical record search proved to be a valid tool for executing this type of register study, where the sample cannot be gathered using only certain medical diagnoses or diagnostic criteria. These type of register pools would be useful also in future studies regarding health implications of doping use.

### Limitations

Our study has several limitations. First, small sample size with great heterogeneity of age and doping use history limits the extrapolation of these findings to all doping using female patients. It should also be recognized that not all adversities linked to doping use require treatment in specialized health care. It is therefore likely that our study does not depict the entire subgroup of females with doping use in Finland, nor does it present the whole spectrum of diseases these patients have. Also, with single hospital district, caution must be applied, as these findings might not be generalizable to all hospital districts in Finland or to other countries. Second, limitations regarding the sampling technique and including only patients who had self-acknowledged doping use has undoubtedly caused exclusion of some patients with doping use and possible selection bias in our study. Third, the follow-up time for each patient could not be calculated. This is because we were not able to track whether these patients had stayed within the HUS catchment area the entire observation period. These patients may also have utilized private health care services, which may in part explain the rarity of some diagnoses. Last, several confounding factors exist (e.g., smoking, diet, medication) causing individual and interindividual variation in red blood cell parameters. Future longitudinal studies with larger number of patients and controlling for such factors are needed to confirm the hematological findings presented in our study. Also, the effect of consecutive AAS use cycles on these parameters warrants investigation.

## Conclusions

The aim of this study was to explore the medical diagnoses of doping using female patients in public specialized health care, the timely relationship between these diagnoses and active doping use, and possible correlation between hematological parameters and the patients’ doping use status. Our findings suggest that female specialized health care patients with current or previous doping use may suffer from high psychiatric and SUD related morbidity. Moreover, patients with active doping use were often referred to specialized health care due to acute medical conditions. In addition, we discovered that red blood cell parameters were within normal limits in majority of AAS using patients, and only minority of the patients in the sample had cardiovascular complications possibly relating to doping use. These results would seem to suggest that the primary method for detecting doping use among female patients in specialized health care should not be based solely on abnormal laboratory parameters or cardiovascular status; instead, acquiring a thorough anamnesis is the cornerstone. Future research is necessary to assess the generalizability of these findings to other populations and to evaluate the psychiatric and somatic comorbidities of these patients in different levels of healthcare. This will help targeting treatment and preventive measures accordingly: at facilities encaged with SUD treatment and mental health care, as well as school health care and youth services, where body modification desires and practices of cis- and transgender adolescents are met. There is reason to believe that the prevailing body image ideal, together with easy availability of drugs via the internet, may increase PIEDs use among women in the future [2]. To outpace “expertise by experience” with professional expertise, more scientific research concentrating on women with doping use will be needed.

## Supplementary Information

Additional file 1. Description of the keywords used for data acquirement.

## Data Availability

The datasets generated and analyzed during the current study are not publicly available due to low number of patients and detailed medical information but are available from the corresponding author on reasonable request.

## Abbreviations

AAS: Anabolic androgenic steroids
APEDs: Appearance- and performance-enhancing drugs
Hb: Hemoglobin concentration
HCT: Hematocrit
HGH: Human growth hormone
HUS: Hospital District of Helsinki and Uusimaa
ICD-10: 10Th revision of the International Statistical Classification of Diseases and Related Health Problems
IGF: Insulin-like growth hormone
IQR: Interquartile range
MCH: Mean corpuscular hemoglobin
MCHC: Mean corpuscular hemoglobin concentration
MCV: Mean corpuscular volume
MSH: Melanocyte-stimulating hormone
PIEDs: Performance and image–enhancing drugs
RBC: Erythrocyte count
RDW: Red cell distribution width
SD: Standard deviation
SUD: Substance use disorder
THL: The Finnish Institute for Health and Welfare
T3: Triiodothyronine
T4: Levothyroxine
