# Supplementary material for: Female doping: observations from a data lake study in the Hospital District of Helsinki and Uusimaa, Finland
Source: BMC Womens Health. 2023 May 9;23:242. doi: 10.1186/s12905-023-02399-9 (PMC10170745; doi:10.1186/s12905-023-02399-9)
Supplement: Supplementary file 1 — Additional file 1. Description of the keywords used for data acquirement. [file 12905_2023_2399_MOESM1_ESM.docx]

# Description of the keywords used for data acquirement

The searched keywords in the HUS data lake’s free text patient records were: “doping*”, “anabol*”, “design-steroid*”; Finnish words “doup*”, “aivodopin*”, “kuntodo*”, “ulkonäködo*”, “steroidido*”; and Finnish words relating to laboratory tests “U-steroid*”, “virtsan steroid*”, “steroidiseulon*”, “U -Anab-O”, “T/E-suh*”. Abbreviations, such as “AAS”, were left out after test search because the consecutive letters as such appear in several Finnish words not relating to doping use. A limitation was set to exclude records with “ei doping*” (in English “no doping*”). To avoid the same text appearing twice in the same context, clinical records from radiology and chemotherapy, as well as referrals to another policlinic, were left out. Also, primarily non-medical records (such as social work) were omitted. Medical certificates for competitive athletes, appointed to the Finnish doping control organization in charge (currently Finnish Center for Integrity in Sports (FINCIS), previously Antidopingtoimikunta ry (ADT)) were minimized by excluding the words “ADT”, “antidoping”; the Finnish word “toimikunta” in connection with “astma” (in English “asthma”) and brand names of two of the most often used medications by athletes that require therapeutic-use exemption (acrivastine and salbutamol).
